# Supplementary material for: Appearances Can Be Deceptive: Morphological, Phylogenetic, and Nomenclatural Delineation of Two Newly Named African Species Related to Frankenia pulverulenta (Frankeniaceae)
Source: Plants (Basel). 2025 Apr 5;14(7):1130. doi: 10.3390/plants14071130 (PMC11991498; doi:10.3390/plants14071130)
Supplement: Supplementary file 1 [file plants-14-01130-s001.zip › plants-3556149-supplementary.pdf]

Supplementary data Fig. S1A–D

Fig. S1A. Maximum Parsimony (MP) 50% consensus tree of *Frankenia* (ITS region)

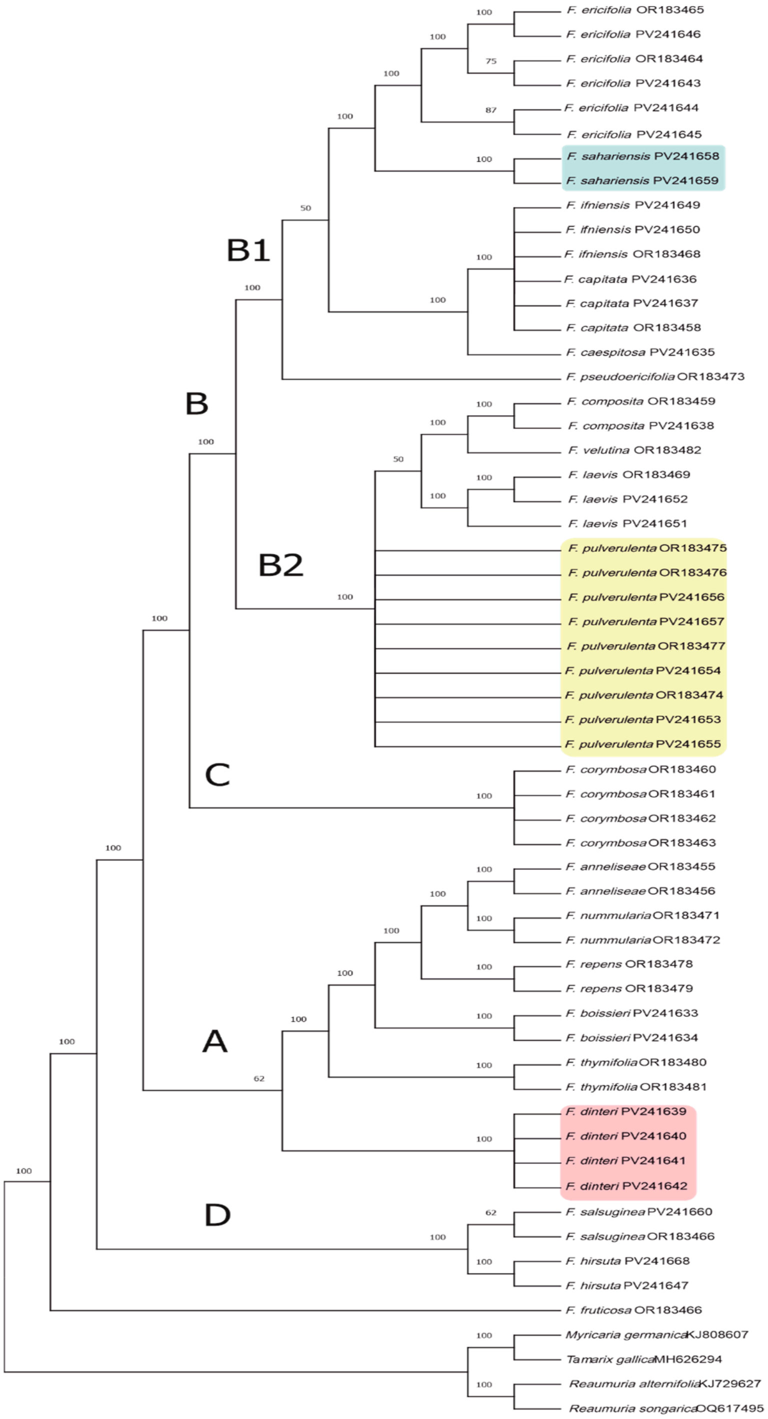

Fig. S1B. Neighbour Joining (NJ) 50% condensed tree of *Frankenia* (ITS region)

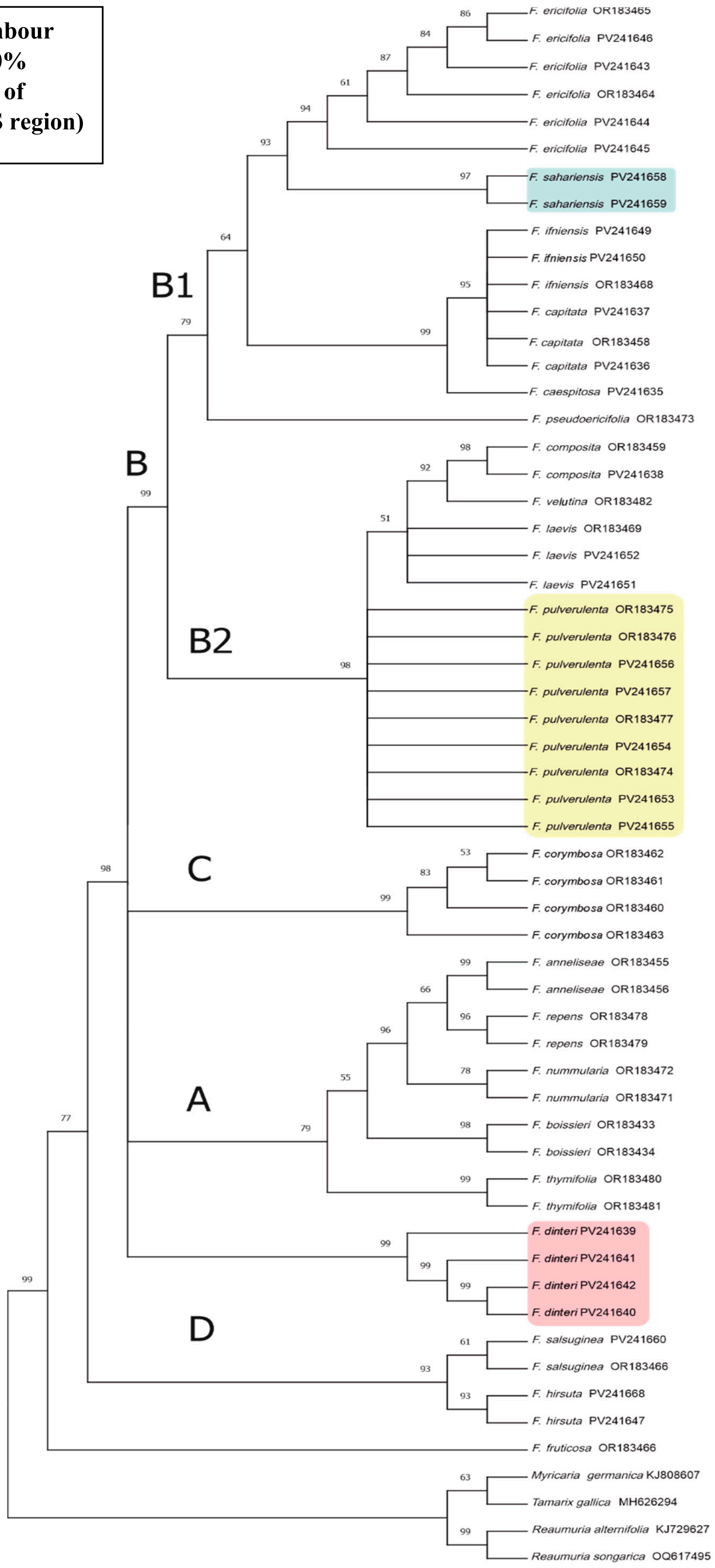

Fig. S1C. Maximum Parsimony (MP) 50% consensus tree of *Frankenia* (*matK* gene)

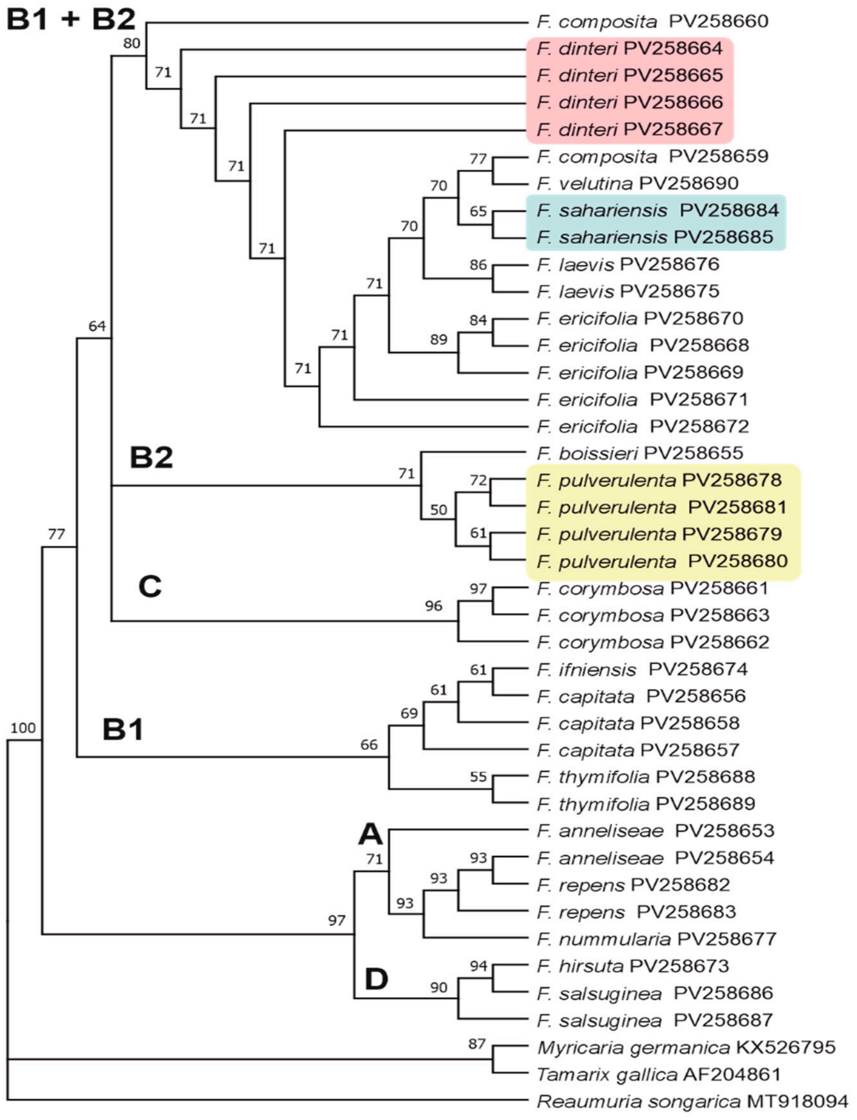

Fig. S1D. Neighbour Joining (NJ) 50% condensed tree of *Frankenia* (*matK* gene)

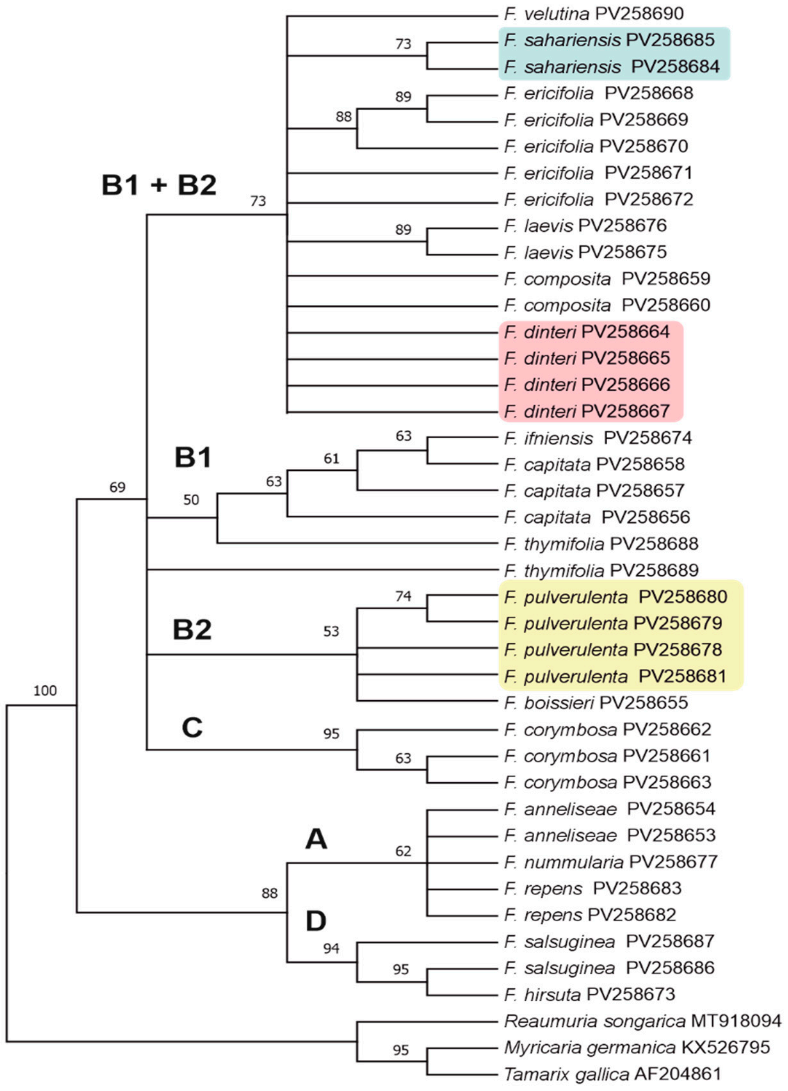

Figure S1. Phylogenetic trees of *Frankenia* accessions as obtained with MEGA (v.10.2.6). The position of *F. sahariensis*, *F. dinteri*, and *F. pulverulenta* is highlighted for easy identification. Numbers above branches indicate bootstrap percentage (BP) values obtained after 10,000 replicates. GenBank codes are shown after each taxon/accession name. A, Maximum Parsimony (MP) 50% consensus tree from ITS nuclear DNA sequences. Eight most parsimonious trees were obtained with a tree length (TL) of 581 steps, a consistency index (CI) of 0.796 and a retention index (RI) of 0.913. **B,** Neighbour Joining (NJ) 50% condensed tree from *matK* plastid DNA sequences. The evolutionary distances were computed using the Kimura 2-parameter method, allowing invariable sites (+*I* = 26.46%). **C,** Maximum Parsimony (MP) 50% consensus tree from *matK* plastid DNA sequences. Nine most parsimonious trees were obtained with a tree length (TL) of 226 steps, a consistency index (CI) of 0.848 and a retention index (RI) of 0.929. **D,** Neighbour Joining (NJ) 50% condensed tree from *matK* plastid DNA sequences. The evolutionary distances were computed using the Tamura 3-parameter (T92) model, coupled with a discrete Gamma distribution (+*G* = 1.0742).
